# Introductory programming course: review and future implications

**DOI:** 10.7717/peerj-cs.647

**Published:** 2021-07-22

**Authors:** Uzma Omer, Muhammad Shoaib Farooq, Adnan Abid

**Affiliations:** 1 Department of Computer Science, University of Management and Technology, Lahore, Punjab, Pakistan; 2 Department of Information Sciences, University of Education, Lahore, Punjab, Pakistan

**Keywords:** Introductory programming, Teaching, Learning, Assessment, Content, Tool, Review

## Abstract

The introductory programming course (IPC) holds a special significance in computing disciplines as this course serves as a prerequisite for studying the higher level courses. Students generally face difficulties during their initial stages of learning how to program. Continuous efforts are being made to examine this course for identifying potential improvements. This article presents the review of the state-of-the-art research exploring various components of IPC by examining sixty-six articles published between 2014 and 2020 in well-reputed research venues. The results reveal that several useful methods have been proposed to support teaching and learning in IPC. Moreover, the research in IPC presented useful ways to conduct assessments, and also demonstrated different techniques to examine improvements in the IPC contents. In addition, a variety of tools are evaluated to support the related course processes. Apart from the aforementioned facets, this research explores other interesting dimensions of IPC, such as collaborative learning, cognitive assessments, and performance predictions. In addition to reviewing the recent advancements in IPC, this study proposes a new taxonomy of IPC research dimensions. Furthermore, based on the successful practices that are listed in the literature, some useful guidelines and advices for instructors have also been reported in this article. Lastly, this review presents some pertinent open research issues to highlight the future dimensions for IPC researchers.

## Introduction

An introductory programming course (IPC) serves to teach the fundamentals of programming in computing disciplines. This course also plays a vital role to build the foundation of subsequent higher level courses in the related study programs. Students mostly face difficulties in learning the basics of computer programming (Watson & Li, 2014). IPC has gained notable attention of researchers who are striving to find any hidden information that could lead to improve the different facets of this course. The research in IPC examined various ways of teaching, learning, and assessments to enhance the relevant aspects (Seeling & Eickholt, 2017; Funabiki, Ishihara & Kao, 2016; Rubio et al., 2014). In addition, the improvisations in contents of IPC have also been investigated in literature (Santana, Figueredo & Bittencourt, 2018; Wainer & Xavier, 2018). Moreover, efforts have also been made to evaluate the use of different tools in order to engage students and support the successful execution of IPC (Pereira et al., 2020; Ureel & Wallace, 2019). The continuous efforts for examining different facets of IPC triggers the need of synthesizing these efforts to find any information that could be utilized to improve the various aspects of this course.

This study aims to investigate different dimensions of IPC that have been examined in research. The motivation of this work is to explore the state-of-the-art trends in IPC and identify the areas for potential improvements. The main objective of this effort is to assist instructors and researchers in their respective domains by critically appraising and summarizing the recent advancements in IPC. This work follows a prescribed technique (Kitchenham & Charters, 2007) to review the existing research in IPC. It presents in-depth examination of the relevant studies through systematic processes of searching, shortlisting, classifying, reviewing, and analyzing the literature.

### Rationale for the review

To the best of our knowledge, few studies reported the review of IPC research. These studies are either anecdotal in nature or related to specific aspects of IPC. A research presented systematic literature review on IPC in higher education (Medeiros, Ramalho & Falcão, 2018). The scope of this study was mainly confined to the teaching and learning approaches in IPC; however, focusing just on these facets does not allow to comprehensively present the IPC aspects that are examined in the literature. Another systematic review was performed to present the comparison of blended learning models that have been applied in IPC (Alammary, 2019). Again, this work demonstrated the analysis of aspects that were related to a particular dimension of IPC. A recent effort performed review on curriculum, teaching and learning, and assessment of IPC (Mehmood et al., 2020). This study presented learning and teaching as a single aspect, which depicts common classification categories; however, our analysis indicated some specific aspects of learning and teaching that need to be highlighted distinctly. Moreover, in this review, we identified and analyzed additional dimensions of IPC, such as cognitive analysis, feedback approaches, collaborative learning techniques, concept specific analysis, predictions, and personalized learning.

A study was conducted to examine the trends in introductory programming (Luxton-Reilly et al., 2018). In this work, the authors categorized the research on the basis of student, teaching, curriculum, and assessment. This study reviewed literature across the breadth, while a substantial part of this research discussed approaches and tools that have been used before the past decade. It was more focused towards discussing the broader aspects as compared to the focus of our work, which is related to the in-depth analysis of the IPC research. Moreover, unlike this previous effort, the analysis of our review is based on a major number of recent studies. This resulted in the identification of additional aspects like robustness of IPC research, course structuring through concept mapping, and behavioral analysis for program profiling.

The above discussion reveals that although some studies attempted to examine IPC research yet these studies either presented the overviews of the trends or evaluated some specific dimensions of IPC. In this work, we aim to present the state-of-the-art trends in IPC by comprehensively examining the different dimensions of IPC research and also highlight some insights for future implications of IPC.

### Contributions and community support

This article presents a new and comprehensive review of the recent advancements in IPC. It is based on examining 66 research articles that are gathered by searching the eminent publication sources. The novelty of this work is the taxonomy that represents 23 different facets of the state-of-the-art research in IPC. Moreover, this study provides a list of advices for IPC instructors to help them conduct the course according to the recent and effectual practices. Lastly, this work highlights the open research issues emerged from analyzing the prominent findings to streamline the future directions for IPC researchers.

The rest of this article has been organized in the following manner. The next section describes the methodology of conducting this review by demonstrating the basic steps of carrying out this work. Then, the results and findings are elaborated by summarizing the extracted outcomes and responses to the research questions. After that, the discussion and analysis are presented, which highlight the principal findings and future implications. Then, the limitations of this review are illustrated. Finally, the conclusions are presented, which outline the main points of this work.

## Methodology

**Figure 1:**
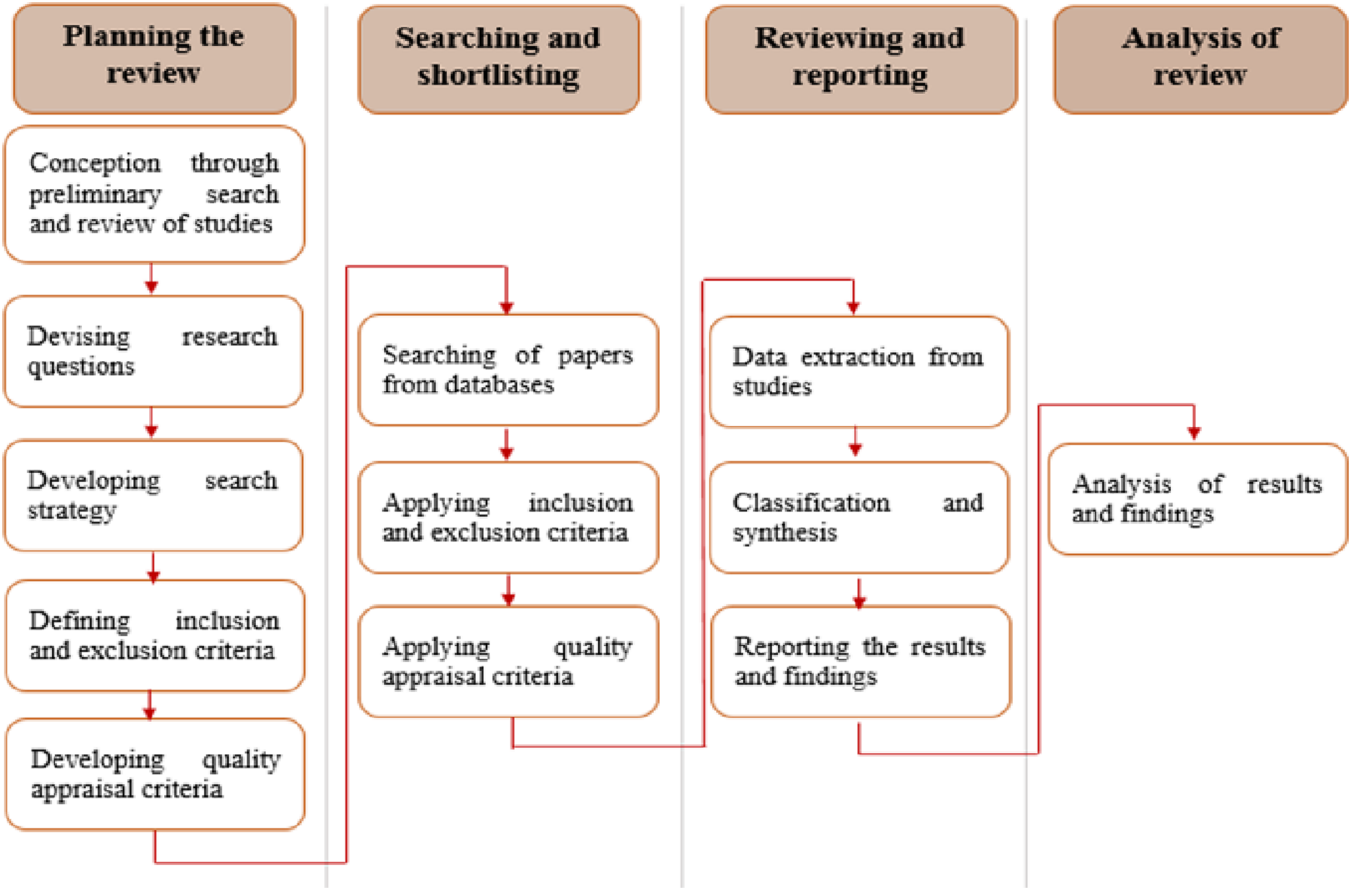
Stages of conducting the review.

As discussed in the “Introduction” section, this review is performed by considering the prescribed guidelines (Kitchenham & Charters, 2007) that include the following major steps: specifying the research questions, performing searches in target databases, selecting and filtering the studies, extracting and synthesizing the data, and reporting. The different stages of performing this review are shown in Fig. 1.

### Planning the review

The planning phase of this review provided the basis for systematically classifying the selected studies and performing the subsequent analysis. This phase has been carried out to establish the conception of the review, devise the research questions, formulate the search strategy, develop the inclusion and exclusion criteria, and define the quality appraisal criteria.

#### Conception of the review

This step has been conducted to establish the basic understanding of the chosen field through preliminary search and scrutiny of the relevant studies. The searching of studies at this stage was performed manually. It resulted in the identification of 24 records. The preliminary analysis of the IPC studies guided to plan and execute the further steps of this review.

#### Research questions (RQs)

Keeping in view the objectives of this study, this review addresses the following RQs to synthesize the recent work in IPC and identify the future implications.

RQ1: Which publication sources are the main targets for IPC research and what different types of research are conducted for IPC?

RQ2: What components of IPC are the areas of focus and in what respects those are examined in IPC studies?

RQ3: What contributions can be perceived on the basis of potential benefits or impacts of IPC research in the field?

#### Search strategy

The search strategy was devised by considering different keywords for searching the relevant records from the digital libraries. These keywords were classified as primary, secondary, and tertiary. The primary set of keywords was established to search the studies, which followed various ways of referring IPC. The secondary set of keywords was devised to find the research articles, which examined different components of IPC, while the tertiary set of keywords was formulated to search the studies, which were specific to student or course. Following is the classification of different keywords that were initially considered for searching the studies:

Primary: Introductory programming, programming fundamentals, programming, CS1

Secondary: learn, teach, assess, content, concept, tool

Tertiary: student, course

The use of logical operators has been emphasized to find the records having the targeted sets of keywords (Marcos-Pablos & García-Peñalvo, 2020). At initial stage of searching the studies, we used two logical operators, AND, and OR, to devise the search strategy. Following was the initial strategy, which involved different classes of keywords and the logical operators to find the relevant papers from the digital libraries: ∀*Pimary* ∧ (∀*Secondary* ∨ ∀*Tertiary*). As a result of executing this approach of searching the studies, a large number of irrelevant records were appeared. Moreover, some databases apply limitations to the number of keywords that can be used in the search string. Hence, we made an optimized selection of keywords to finalize the search string with the intent of targeting more relevant records. From the primary set of keywords, we used “introductory programming”, “programming fundamentals”, and “CS1”, as these were appeared to be the most commonly used terms for representing IPC in literature. Through these keywords, we aimed to search the studies that were related to IPC or the research of programming courses that analyzed specific IPC concepts. From secondary and tertiary sets of keywords, we selected “course” and “student” to target the studies that demonstrated course or student related findings and were focused on different areas of IPC. Moreover, the previous searches showed some irrelevant records that were related to the higher level programming courses like visual or windows programming. Hence, we used another logical operator, NOT, to reduce the possibility of appearing such irrelevant records in the search results. Table 1 enlists the search strings that were specifically applied to each digital repository. Following was the generic search string for searching the relevant papers.

**Table 1:** Search strings with respect to the digital repositories.

Database	Search string
IEEE Xplore	(“Introductory programming” OR “Programming fundamentals” OR “CS1”) AND (“course” OR “student”) NOT (“Visual programming” OR “Windows programming”)
ACM Digital library	[[All: “introductory programming”] OR [All: “cs1”]] AND [[All: “course”] OR [All: “learn”]] AND NOT [[All: “visual programming”] OR [All: “windows programming”]]
SpringerLink	‘(“Introductory programming” OR “Programming fundamentals” OR “CS1”) AND (“course” OR “student”) NOT (“Visual programming” OR “Windows programming”)’
Wiley	“(“Introductory programming” OR “Programming fundamentals” OR “CS1”) AND (“course” OR “student”) NOT (“Visual programming” OR “Windows programming”)”
Google Scholar	(“Introductory programming” OR “Programming fundamentals” OR “CS1”) AND (“course” OR “student”) NOT (“Visual programming” OR “Windows programming”)
Taylor and Francis	**[[All: “introductory programming”] OR [All: “programming fundamentals”] OR [All: “cs1”]] AND [[All: “course”] OR [All: “student”]] AND NOT [[All: “visual programming”] OR [All: “windows programming”]]**
MDPI	(“Introductory programming” OR “Programming fundamentals” OR “CS1”) AND (“course” OR “student”) NOT (“Visual programming” OR “Windows programming”)
ERIC	(“Introductory programming” OR “Programming fundamentals” OR “CS1”) AND (“course” OR “student”) NOT (“Visual programming” OR “Windows programming”)
SAGE	[[All “introductory programming”] OR [All “programming fundamentals”] OR [All “cs1”]] AND [[All “course”] OR [All “student”]] AND NOT [[All “visual programming”] OR [All “windows programming”]]
Scopus	“Introductory Programming” OR “Programming fundamentals” OR “CS1” AND “course” AND “student” AND NOT “Virtual programming” AND NOT “Windows programming”

(“Introductory programming” OR “Programming fundamentals” OR “CS1”) AND (“course” OR “student”) NOT (“Visual programming” OR “Windows programming”)

The databases for searching the relevant papers were identified during the preliminary analysis of the literature. Field specific and the most prominent research venues were selected to find the relevant studies. The searches were conducted in the following publication sources: Scopus, ACM Digital Library, IEEE Xplore, SpringerLink, Wiley, Taylor and Francis, MDPI, SAGE, and ERIC. After searching the papers in these sources, a search was performed on google scholar to find any relevant paper that may have left from the prior searches.

#### Inclusion and exclusion criteria

During the preliminary analysis of papers, some records with inappropriate scopes and focuses (as per the investigating areas of this research) were identified. This further guided to establish the inclusion and exclusion criteria for screening the most relevant records from the search results. Table 2 presents the inclusion and exclusion criteria to shortlist the studies for this review.

**Table 2:** Inclusion and exclusion criteria.

Inclusion Criteria (IC)	**IC1**	Studies representing teacher and student-centric environments that are related to formal education system.
	**IC2**	Empirically validated studies.
	**IC3**	Papers that are focused on introductory programming course of higher level education.
Exclusion Criteria (EC)	**EC1**	Research examining holistic aspects such as examining overall study programs.
	**EC2**	Papers not having concrete validation of the proposed solution/techniques, such as opinion papers, future directions, and reviews.
	**EC3**	Studies that focused on introductory programming course at school level.
	**EC4**	Papers that are not in English.

#### Quality appraisal criteria

The quality appraisal of the selected studies was performed on the basis of the following criteria as defined in a previous research (Ouhbi et al., 2015): (a) solution is well defined and possesses methodical potential; (b) conclusion reflects findings; (c) methodology is clear and well defined; and (d) publication source is stable and well-reputed. The studies for criteria ‘a’, ‘b’, and ‘c’ were ranked on the basis of following scores: 1 for fulfilling the respective criteria, 0 for not fulfilling, and 0.5 for partially fulfilling the respective criteria. Criterion ‘d’ was rated by considering the Computer Science Conference rankings (CORE), and the Journal Citation Reports (JCR) lists. Table 3 illustrates the possible ratings for scoring the selected studies for criterion ‘d’.

**Table 3:** Possible ratings of publication sources.

Journal papers ranks scoring				Others (No JCR ranking)	Conference papers ranks scoring				Others (No CORE ranking)
Q1	Q2	Q3	Q4		Core A*	Core A	Core B	Core C	
2	1.5	1	0.5	0	1.5	1	0.75	0.5	0

### Searching and shortlisting the papers

**Figure 2:**
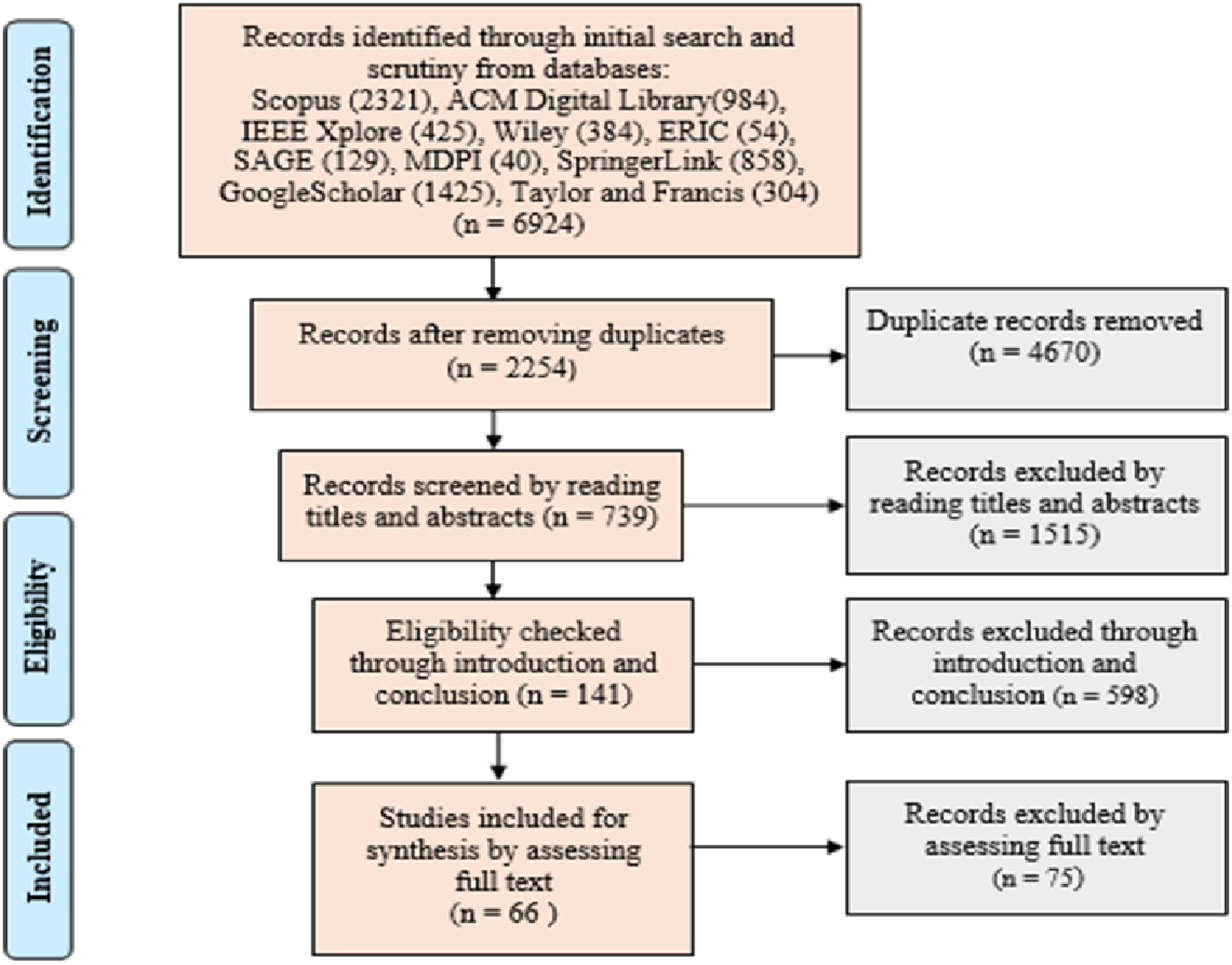
Shortlisting process of studies.

The studies obtained after applying the search strings to the databases were further scrutinized for shortlisting. The scrutiny of studies at this stage was performed on the basis of year-wise filtration. Moreover, the research published as technical report, thesis, or the work that did not reflect relevancy according to our research area, was also excluded. The shortlisting process of the studies, after performing the initial scrutiny from databases, is presented in Fig. 2.

The duplicate records were excluded at first stage of screening the identified papers. After this stage, the papers were shortlisted by reading the titles and abstracts. Then, the screening was performed on the basis of the introduction and conclusion sections. Lastly, the studies were shortlisted through full text assessment. This resulted in selection of 66 papers for this systematic review. The final round of searching the research articles was conducted in October 2020.

### Data extraction and classification

The data was extracted from the selected studies on the basis of the aspects that were inquired through the RQs.

RQ1 was defined to explore the bibliometric facts and types of research. The bibliometric facts can be classified according to the years and sources of publications, while a type of research can be classified into the following categories as suggested in a previous work (Ouhbi et al., 2015): (1) model, which provides representation of a system by linking related aspects; (2) framework, which presents a real or conceptual structure that guides the expansion of the structure to solve the identified problems; (3) improvement, which modifies existing work to evaluate betterments in the outcomes; (4) experiment, which expresses the personal experience of authors and presents an empirical method under controlled conditions; (5) case study, which examines an empirical inquiry related to specific context; and (6) evaluation, which provides the comparison of similar facets of IPC.

RQ2 was formulated to examine the areas of focus in IPC studies. An area of focus can mainly be classified into the following categories: (1) teaching, which investigates one or more teaching techniques; (2) learning, which scrutinizes the learning approaches; (3) tool, which evaluates one or more features of a system; (4) assessment, which checks the effectiveness of some assessment techniques or material; and (5) content, which primarily examines course concepts or programming languages.

**Figure 3:**
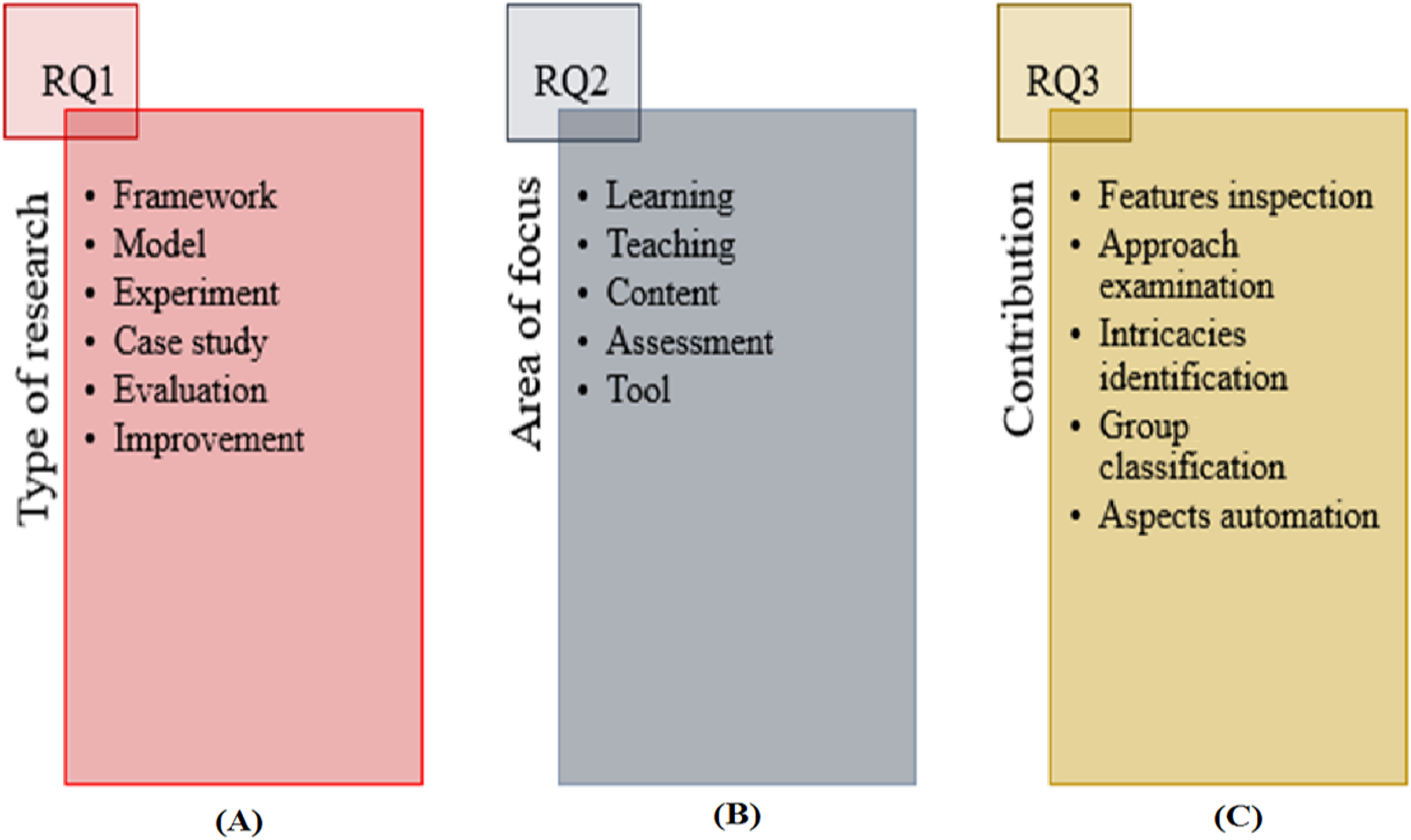
Classification with respect to RQs.

RQ3 was devised to investigate the perceived contributions on the basis of the potential impacts or benefits of the IPC research in the field. A perceived contribution can be classified into the following categories: (1) feature inspection, which discovers different parameters that can be utilized to examine or predict students’ performance; (2) approach examination, which presents the findings by analyzing different teaching, learning or analysis approaches; (3) intricacies identification, which identifies difficulties in comprehending different components of course; (4) group classification, which contributes by categorizing particular types of facets based on specific criteria; and (5) aspects automation, which demonstrates the automation of some specific aspect of IPC. Figure 3 shows the defined classification with respect to RQ1 (A), RQ2 (B) and RQ3(C).

## Results and findings

### Quality assessment

Table 4 presents the quality assessment of the shortlisted articles. The details of scoring are shown in Table 13. Major difference in scoring is appeared due to the quality criterion ‘d’. The criteria ‘a’, ‘b’ and ‘c’ have been addressed in most of the shortlisted studies.

**Table 4:** Quality assessment of shortlisted studies.

Studies	Scoring classification	Total
Liao et al., 2019; Yeomans, Zschaler & Coate, 2019; Carter, Hundhausen & Adesope, 2017; Iqbal Malik & Coldwell-Neilson, 2017; Pereira et al., 2020	Exactly 5	5
McCall & Kölling, 2019; Ullah et al., 2019; Ureel & Wallace, 2019; Albluwi, 2018; Bhatia, Kohli & Singh, 2018; Lagus et al., 2018; Lin et al., 2018; Turner, Pérez-Quiñones & Edwards, 2018; Wainer & Xavier, 2018; Castro-Wunsch, Ahadi & Petersen, 2017; Ahadi, Hellas & Lister, 2017; Echeverría et al., 2017; Hsiao, Huang & Murphy, 2017; Malliarakis, Satratzemi & Xinogalos, 2016; Scott et al., 2015; Xinogalos, 2015; Allinjawi, Al-Nuaim & Krause, 2014; Omer, Farooq & Abid, 2020	Greater or equal to 4 and less than 5	18
Azcona, Hsiao & Smeaton, 2018; Esteero et al., 2018; Gomes & Correia, 2018; King, 2018; Koong et al., 2018; Premchaiswadi, Porouhan & Premchaiswadi, 2018; Dorodchi, Dehbozorgi & Frevert, 2017; España-Boquera et al., 2017; Estey, Keuning & Coady, 2017; Fu et al., 2017; Kumar, 2017; Seanosky et al., 2017; Seeling & Eickholt, 2017; Gomes, Correia & Abreu, 2016; Seeling, 2016; Simkins & Decker, 2016; Ahadi et al., 2015; Ashenafi, Riccardi & Ronchetti, 2015; Carter, Hundhausen & Adesope, 2015; Hilton & Rague, 2015; Janke, Brune & Wagner, 2015; Rosiene & Rosiene, 2015; Su et al., 2015; Ureel & Wallace, 2015; Wood & Keen, 2015; Edwards, Shams & Estep, 2014; Edwards, Tilden & Allevato, 2014; Heinonen et al., 2014, Hijon-Neira et al., 2014; Rubio et al., 2014; Watson, Li & Godwin, 2014; Zur & Vilner, 2014; Ninrutsirikun et al., 2020	Greater or equal to 3 and less than 4	33
Chaweewan et al., 2018; Santana, Figueredo & Bittencourt, 2018; Ahmad et al., 2017; al-Rifaie, Yee-King & d’Inverno, 2017; Delev & Gjorgjevikj, 2017; Berges et al., 2016; Funabiki, Ishihara & Kao, 2016; Doshi, Christian & Trivedi, 2014; Chung & Hsiao, 2020; Effenberger, Pelánek & Čechák, 2020	Greater than 2 and less than 3	10

### Year-wise publication trends

**Figure 4:**
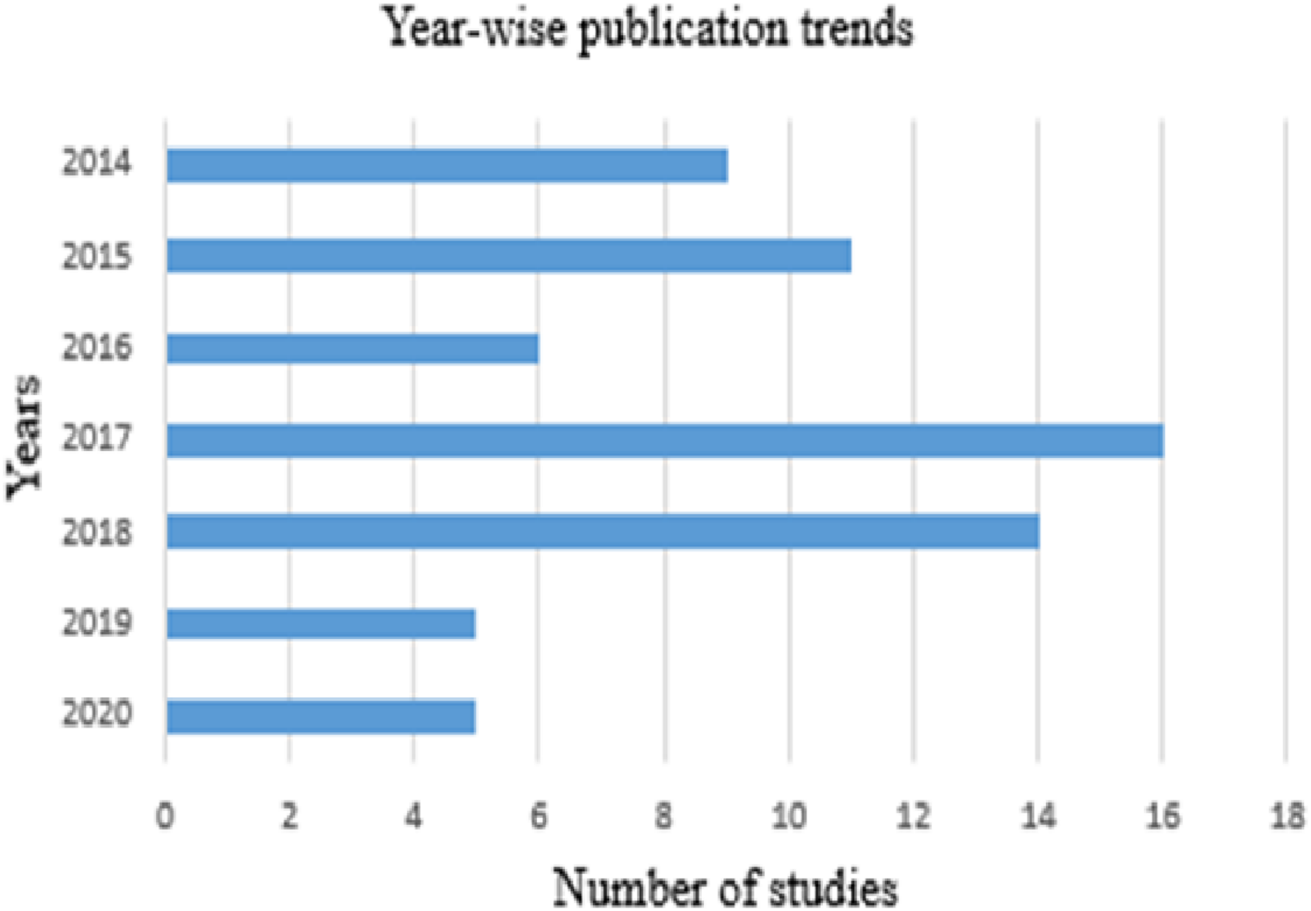
Year-wise trends of IPC research.

The largest number of the shortlisted articles were published in 2017, which makes about 27% of the selected studies. About 24% of the shortlisted studies were appeared in each of the years 2016 and 2018. Around 17% of these studies were emerged in 2015, 14% were published in 2014, and about 8% were produced in 2019. Only 4% of these papers were published in the year 2020. This could be because of the reason that the data collection for this study was ended in October 2020. Therefore, the percentage of papers presented for 2020 may not depict the precise picture of the whole year. Figure 4 shows the year-wise trends of IPC publications.

### RQ1: Which publication sources are the main targets for IPC research and what different types of research are conducted for IPC?

Table 5 presents the publication sources and the channels of the research articles selected for this review. The journals and conferences were identified as the two publication channels. About 32% of the shortlisted studies were published in journals, while about 68% of these studies were presented at conferences.

**Table 5:** Publication sources and channels of selected studies.

Source	Studies	Channel	Count	%
IEEE Frontiers in Education Conference	Azcona, Hsiao & Smeaton, 2018; Gomes & Correia, 2018; King, 2018; Santana, Figueredo & Bittencourt, 2018; Dorodchi, Dehbozorgi & Frevert, 2017; Kumar, 2017; Seeling & Eickholt, 2017; Gomes, Correia & Abreu, 2016; Zur & Vilner, 2014; Seeling, 2016; Simkins & Decker, 2016; Ashenafi, Riccardi & Ronchetti, 2015; Hilton & Rague, 2015; Rubio et al., 2014; Rosiene & Rosiene, 2015; Su et al., 2015; Ureel & Wallace, 2015; Heinonen et al., 2014	C	18	27.27
ACM Transactions on Computing Education	Liao et al., 2019; McCall & Kölling, 2019; Yeomans, Zschaler & Coate, 2019; Lagus et al., 2018; Turner, Pérez-Quiñones & Edwards, 2018; Wainer & Xavier, 2018; Ahadi, Hellas & Lister, 2017; Carter, Hundhausen & Adesope, 2017; Xinogalos, 2015; Allinjawi, Al-Nuaim & Krause, 2014	J	10	15.15
ACM Special Interest Group on Computer Science Education	Ureel & Wallace, 2019; Esteero et al., 2018; Castro-Wunsch, Ahadi & Petersen, 2017; Estey, Keuning & Coady, 2017; Watson, Li & Godwin, 2014; Wood & Keen, 2015; Edwards, Shams & Estep, 2014; Edwards, Tilden & Allevato, 2014; Heinonen et al., 2014	C	10	15.15
IEEE Transactions on Education	Albluwi, 2018; Lin et al., 2018; Scott et al., 2015	J	3	4.55
ACM/IEEE International Conference on Software Engineering	Bhatia, Kohli & Singh, 2018; Janke, Brune & Wagner, 2015	C	2	3.03
ACM International Conference on International Computing Education Research	Ahadi et al., 2015; Carter, Hundhausen & Adesope, 2015	C	2	3.03
International Conference on Learning Analytics & Knowledge	Fu et al., 2017; Effenberger, Pelánek & Čechák, 2020	C	2	3.03
IEEE Transactions on Emerging Topics in Computing	Hsiao, Huang & Murphy, 2017	J	1	1.52
IEEE Transactions on Learning Technologies	Malliarakis, Satratzemi & Xinogalos, 2016	J	1	1.52
IEEE ACCESS	Ullah et al., 2019	J	1	1.52
Computer Applications in Engineering Education Wiley	Echeverría et al., 2017	J	1	1.52
Journal of Educational Computing Research SAGE	Iqbal Malik & Coldwell-Neilson, 2017	J	1	1.52
Wireless Personal Communications	Ninrutsirikun et al., 2020	J	1	1.52
British Journal of Educational Technology	Pereira et al., 2020	J	1	1.52
Sustainability	Omer, Farooq & Abid, 2020	J	1	1.52
IEEE International Conference on Computer Software & Applications	Koong et al., 2018	C	1	1.52
IEEE Computer Software and Applications Conference	Premchaiswadi, Porouhan & Premchaiswadi, 2018	C	1	1.52
IEEE International Conference on Advanced Learning Technologies	Seanosky et al., 2017	C	1	1.52
IEEE International Conference on IT Based Higher Education and Training	España-Boquera et al., 2017	C	1	1.52
IEEE International Conference on Computer and Communication Systems	Chaweewan et al., 2018	C	1	1.52
IEEE World Engineering Education Forum	Ahmad et al., 2017	C	1	1.52
IEEE Intelligent Systems Conference	al-Rifaie, Yee-King & d’Inverno, 2017	C	1	1.52
IEEE Global Engineering Education Conference	Delev & Gjorgjevikj, 2017	C	1	1.52
IEEE International Conference on Learning and Teaching in Computing and Engineering	Berges et al., 2016	C	1	1.52
IEEE Global Conference on Consumer Electronics	Funabiki, Ishihara & Kao, 2016	C	1	1.52
IEEE international conference on technology for education	Doshi, Christian & Trivedi, 2014	C	1	1.52

**Note:**

J is abbreviated for journal and C for conference.

Most of the journal articles were published in ACM Transactions on Computing Education (TOCE), while a large number of conference papers were presented at IEEE Frontiers in Education (FIE). Other prominent sources of the shortlisted studies include: ACM/IEEE International Conference on Software Engineering (ICSE), ACM Special Interest Group on Computer Science Education (SIGCSE), IEEE Transactions on Education (TOE), ACM International Conference on International Computing Education Research (ICER), and IEEE International Conference on Computer Software and Applications (COMPSAC).

Table 6 presents the classification of studies according to the different types of research. As discussed in the “Data extraction and classification” section, six major types of research were identified. These types are further explained by discussing some examples.

**Table 6:** Classification of types of research.

Types	Studies
Model	Liao et al., 2019; Bhatia, Kohli & Singh, 2018; Ninrutsirikun et al., 2020; Effenberger, Pelánek & Čechák, 2020; Iqbal Malik & Coldwell-Neilson, 2017; Ashenafi, Riccardi & Ronchetti, 2015; Carter, Hundhausen & Adesope, 2015
Framework	Yeomans, Zschaler & Coate, 2019; Chaweewan et al., 2018; Omer, Farooq & Abid, 2020; Premchaiswadi, Porouhan & Premchaiswadi, 2018
Experiment	McCall & Kölling, 2019; Ullah et al., 2019; Ureel & Wallace, 2019; Pereira et al., 2020; Esteero et al., 2018; Gomes & Correia, 2018; King, 2018; Lin et al., 2018; Turner, Pérez-Quiñones & Edwards, 2018; Ahadi, Hellas & Lister, 2017; Ahmad et al., 2017; Delev & Gjorgjevikj, 2017; Dorodchi, Dehbozorgi & Frevert, 2017; Echeverría et al., 2017; España-Boquera et al., 2017; Estey, Keuning & Coady, 2017; Chung & Hsiao, 2020; Fu et al., 2017; Hsiao, Huang & Murphy, 2017; Kumar, 2017; Seanosky et al., 2017; Berges et al., 2016; Funabiki, Ishihara & Kao, 2016; Gomes, Correia & Abreu, 2016; Malliarakis, Satratzemi & Xinogalos, 2016; Zur & Vilner, 2014; Seeling, 2016; Ahadi et al., 2015; Rubio et al., 2014; Rosiene & Rosiene, 2015; Scott et al., 2015; Ureel & Wallace, 2015; Wood & Keen, 2015; Xinogalos, 2015; Allinjawi, Al-Nuaim & Krause, 2014; Doshi, Christian & Trivedi, 2014; Edwards, Shams & Estep, 2014; Edwards, Tilden & Allevato, 2014; Heinonen et al., 2014
Improvement	Azcona, Hsiao & Smeaton, 2018; Koong et al., 2018; Lagus et al., 2018; Carter, Hundhausen & Adesope, 2017; Castro-Wunsch, Ahadi & Petersen, 2017; Su et al., 2015; Hijon-Neira et al., 2014
Case study	Albluwi, 2018; Santana, Figueredo & Bittencourt, 2018; al-Rifaie, Yee-King & d’Inverno, 2017; Simkins & Decker, 2016; Janke, Brune & Wagner, 2015
Evaluation	Wainer & Xavier, 2018; Seeling & Eickholt, 2017; Watson, Li & Godwin, 2014; Hilton & Rague, 2015

A framework was proposed for detailed examination of students’ understanding of various concepts to identify the troublesome concepts (Yeomans, Zschaler & Coate, 2019). The time spent on performing various course related activities has been observed as a commonly utilized parameter to analyze learning in framework-based research (Chaweewan et al., 2018; Premchaiswadi, Porouhan & Premchaiswadi, 2018). The case studies were conducted, e.g., to check the effect of altering the content sequence (Janke, Brune & Wagner, 2015) and to identify the impact of teaching different programming languages on learning (Santana, Figueredo & Bittencourt, 2018). Improvement related work presented refinements on already conducted research. A study presented refinements in the previously proposed technique that used the programming data to analyze learning (Carter, Hundhausen & Adesope, 2017). The refined version of this technique additionally used the social learning behavioral data to examine students’ performance. The evaluation of IPC aspects was performed, e.g., by comparing different pedagogies and analyzing their effect on learning (Seeling & Eickholt, 2017). The models were proposed in the selected studies as solutions to the specified problems. A study presented a model to analyze learning and predict students’ performance (Liao et al., 2019). Similarly, another model was proposed to systematically identify errors in students’ programs (Bhatia, Kohli & Singh, 2018). Experiments were performed to investigate various facets of IPC, which include difficulties of learning (Gomes & Correia, 2018; Xinogalos, 2015) and effectiveness of specific assessment techniques (King, 2018).

### RQ2: What components of IPC are the areas of focus and in what respects those are examined in IPC studies?

**Figure 5:**
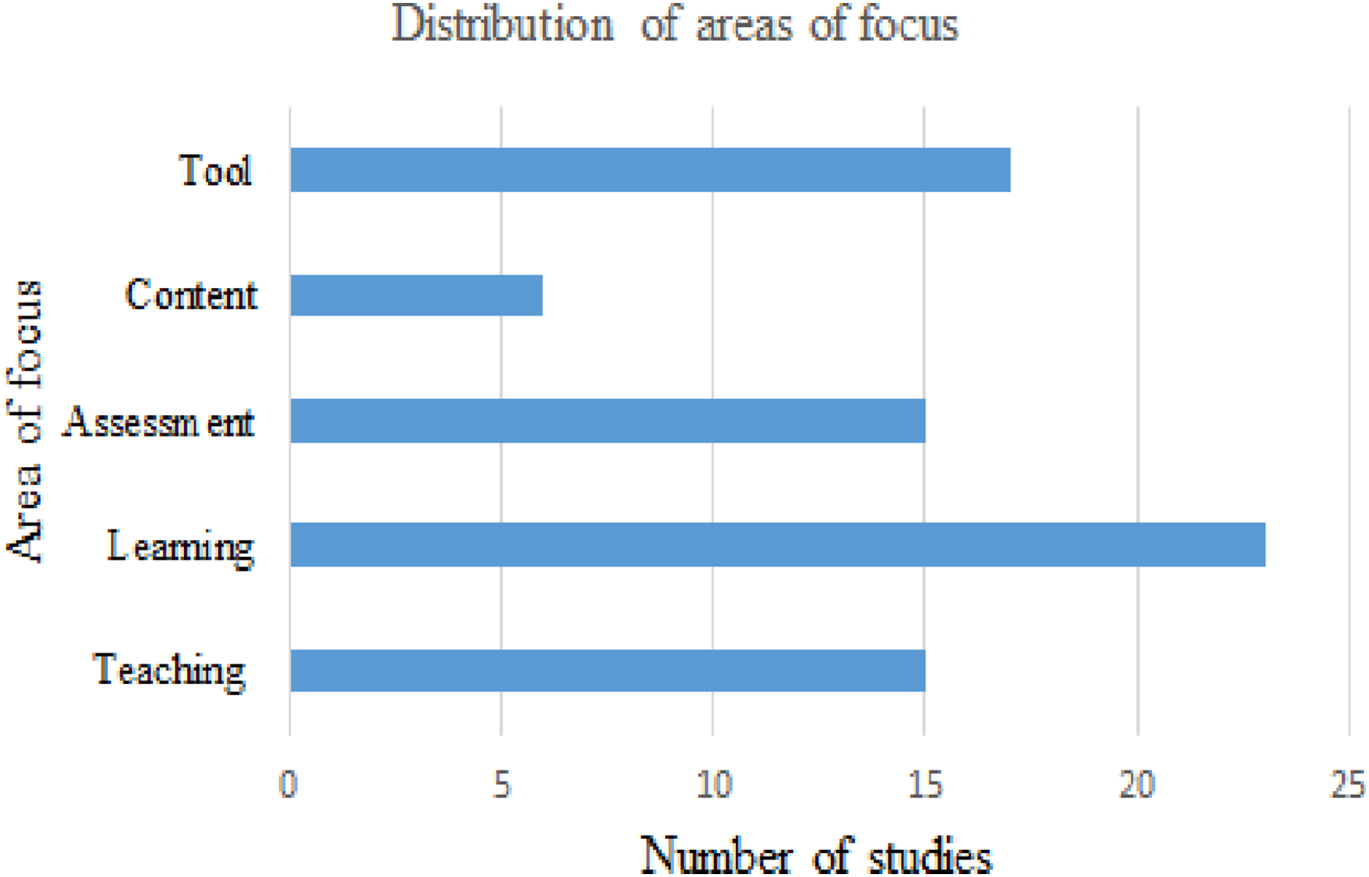
Studies focused on different areas of IPC.

The classification of selected studies on the basis of areas of focus revealed five components of IPC that are mainly examined in IPC research. These components are teaching, learning, assessment, content, and tool. Figure 5 shows the number of studies according to the major areas of focus. The focuses of some studies relate to more than one component of IPC. About 35% of the selected studies were focused on learning approaches, while 26% of these studies inspected the usefulness of various tools. Each of the teaching and assessment dimensions were examined in about 23% of the shortlisted studies, while the contents were analyzed in about 11% of these studies.

**Figure 6:**
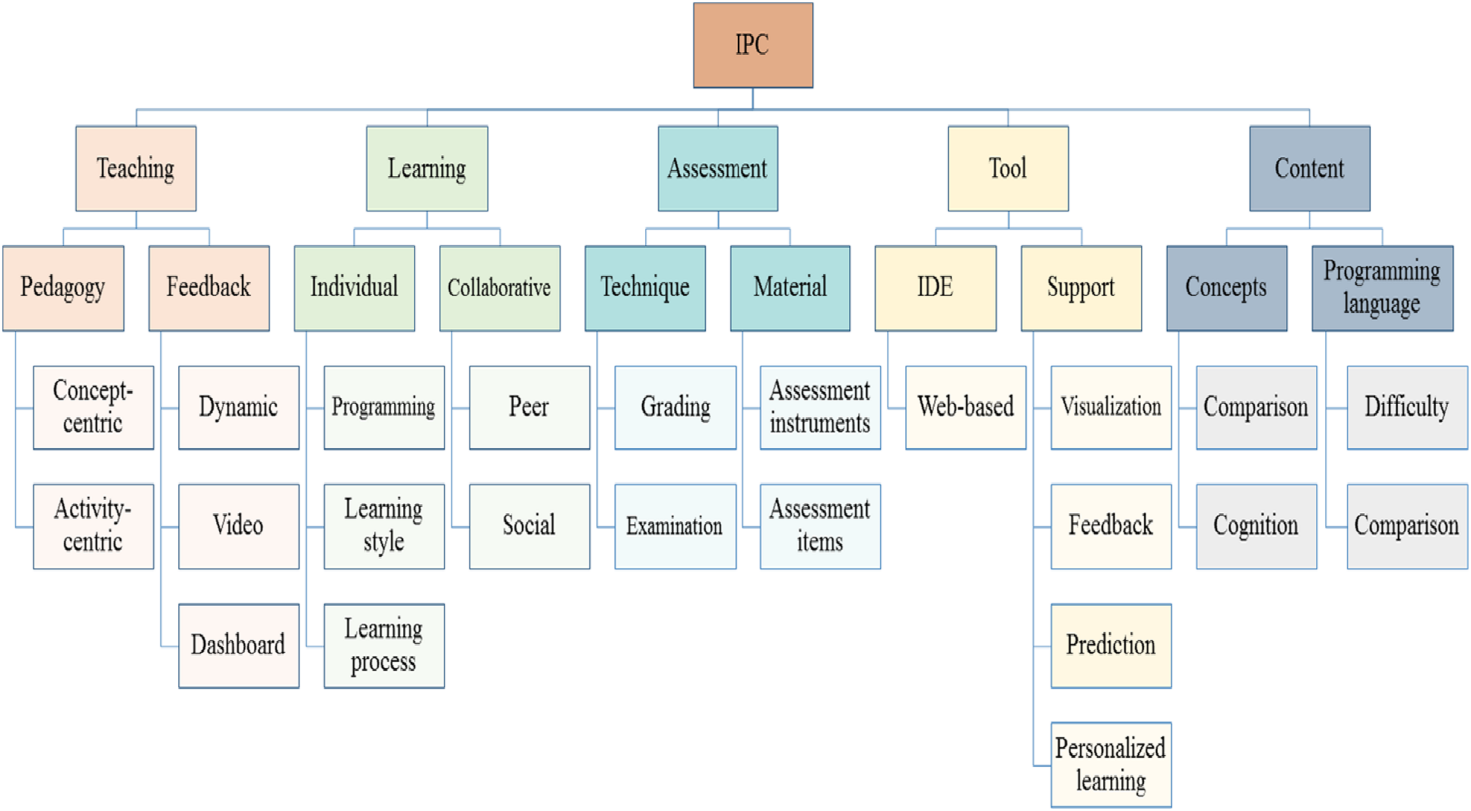
Taxonomy of introductory programming course aspects.

The areas of focus are further classified to present the taxonomy of IPC research as shown in Fig. 6. The first level of the taxonomy presents the main components, which exhibit the major dimensions of IPC studies. These dimensions are further categorized to demonstrate the precise classification by representing different sub-dimensions. The teaching is investigated through different pedagogies and feedback techniques, while the learning is explored on the basis of individual and collaborative learning approaches. The contents are examined by evaluating the specific concepts and the programming languages that are used to teach IPC. The assessment has been scrutinized through different assessment techniques and assessment material, while a tool is inspected as support tool or integrated development environment (IDE). Further description of the major areas of focus and the associated sub-dimensions are summarized in the following part of this section.

#### Teaching

The teaching related aspects were explored by analyzing different pedagogies. The pedagogies can be classified into concept-centric or activity-centric. The concept-centric classification presents those studies in which the pedagogies are analyzed by altering the sequence of teaching programming concepts. The activity-centric pedagogies are explored either by changing the sequence of course related activities or by scrutinizing the impact of conducting one or more activity on students’ learning. In addition, the teaching approaches were investigated through different feedback techniques, which include the dynamic feedback, video-based feedback, and the feedback that was augmented with the visualization of students’ performance through dashboards. Table 7 illustrates brief descriptions of the teaching aspects that were focused in the selected studies and the respective findings.

**Table 7:** Teaching focused research.

Leaf node categories	Brief description of major area of focus	Brief description of major findings	Articles
**Pedagogy**			
Concept-centric	Teaching object oriented concepts first as opposed to following the traditional sequence of teaching IPC concepts.	No significant differences in students’ performances.	Janke, Brune & Wagner, 2015
	Physical computing modules to teach programming concepts.	Enhanced students’ motivation towards learning.	Rubio et al., 2014
Activity-centric	Altering the activity sequence by following the sequence of approach, deployment, result, and improvement.	Positive impact on students' learning and final outcomes.	Iqbal Malik & Coldwell-Neilson, 2017
	Pedagogies comparisons to examine pedagogies based on different sequence of activities.	Active learning pedagogy approach resulted better outcomes.	Seeling & Eickholt, 2017
	Empowered students to plan and schedule the course related activities themselves.	Improvements in students’ performance.	Seeling, 2016
	Examined the impact of the flip - classroom approach.	Identified good, bad and worst aspects of flip-classroom approach.	Rosiene & Rosiene, 2015
	Robot Olympics, as an activity that was based on first code and then refinement of the code.	Improvements in students’ performance.	Scott et al., 2015
	Examined the impact of playing multi player online game.	Better performance as compared to the controlled group.	Malliarakis, Satratzemi & Xinogalos, 2016
	Virtual worlds project to bridge gap between imperative and object oriented paradigm.	Identified activities to design the learning process.	Wood & Keen, 2015
**Feedback**			
Dynamic	Reporting anti-patterns in students’ programs.	Immediate feedback by evaluating students’ programs.	Ureel & Wallace, 2019
	Feedback using test-driven technique.		Ureel & Wallace, 2015
Video	Video feedback technique in comparison to written feedback.	Students preferred video feedback over written feedback.	Hilton & Rague, 2015
Dashboard	Analysis of programming and learning behaviors presented through dashboard.	Improvement in the process of feedback deliverance by providing insight into students’ learning.	Fu et al., 2017
	Visual feedback and its effect on students’ performance.	Students’ performances were not improved through visual feedback without system interactions.	Seanosky et al., 2017

#### Summary

A number of different pedagogies were examined in the selected studies. Gamification was deployed as an activity to help students understand programming. Teaching the programming concepts by linking those with real world objects could support learners to conceive the notions behind different programming concepts. Most of the pedagogies were learner-centric in which the learners were required to perform different activities like implementing a specific project and scheduling the learning activities. A few of the pedagogies were teacher-centric through which different teaching techniques were examined. Feedback approaches were mostly tool oriented in which the tools were used to facilitate the feedback delivery processes. The timing of feedback is significant to identify and address the learning gaps. In this context, the dynamic feedback could be useful to provide real time analysis of the learning states and to plan appropriate interventions.

#### Learning

Learning approaches were further categorized into individual and collaborative learning. The sub-categories of collaborative learning include peer and social learning, while the sub-categories of individual learning include programming, learning style, and learning process. We differentiated the learning styles from learning processes on the basis of providing learners leverage of developing their own pace and style of learning in the case of the former. The learning processes were investigated by examining learners on the basis of specific and defined processes of learning. Table 8 enlists brief descriptions of the learning focused approaches in the selected studies along with the respective findings.

**Table 8:** Learning focused research.

Leaf node categories	Brief description of major area of focus	Brief description of major findings	Articles
**Individual**			
Programming	Severity of errors to identify learning difficulties.	Identification of difficult to fix errors to plan appropriate interventions.	McCall & Kölling, 2019
	Use of syntactically correct programs to automatically correct buggy programs.	Correction of errors in programs.	Bhatia, Kohli & Singh, 2018
	Programming profiles to identify the aptitudes and skills.	Programming profiles helped instructors to guide students.	Chaweewan et al., 2018
	Static analysis of students’ codes to find common occurring errors.	Identification of most frequent errors.	Delev & Gjorgjevikj, 2017
	Scrutinizing the errors in students' programs.	Identification of missing competencies.	Berges et al., 2016
	Identification of non-terminating code.	Indication of the problematic parts of the code.	Edwards, Shams & Estep, 2014
	Parameters and techniques to analyze learning or predict performance.	Identification of programming parameters or techniques that effect students’ performance.	Lagus et al., 2018; Ninrutsirikun et al., 2020; Castro-Wunsch, Ahadi & Petersen, 2017; Ahadi, Hellas & Lister, 2017; Watson, Li & Godwin, 2014; Ahadi et al., 2015; Ashenafi, Riccardi & Ronchetti, 2015; Carter, Hundhausen & Adesope, 2015
Learning styles	Learning styles and their effect on outcomes.	Identification of learning styles that resulted in better outcomes.	Kumar, 2017
	The relationships of micro and macro learning patterns with final performance.	Patterns demonstrated better correlation for good performances.	Chung & Hsiao, 2020
	Students’ engagements in course related activities, to predict performance.	Examined the features to predict students’ performance.	Premchaiswadi, Porouhan & Premchaiswadi, 2018
Learning process	Learning difficulties and their causes.	Identification of learning difficulties and their potential causes.	Simkins & Decker, 2016
	Genetic algorithm to identify personal learning needs.	Identification of personal learning needs of students.	Lin et al., 2018
**Collaborative**			
Peer	Peer instruction for collaborative learning.	Established relationship between students’ performance and collaborative learning technique.	Liao et al., 2019
	Peer feedback on programming.	Positive effect on learning and students’ performance.	Azcona, Hsiao & Smeaton, 2018
Social	Social learning activities to predict students’ performances.	Cumulative activities reflected better accuracies than individual activities.	al-Rifaie, Yee-King & d’Inverno, 2017
	Social learning behavior along with the programming behavior for prediction.	Prediction accuracies improved with social learning behavior.	Carter, Hundhausen & Adesope, 2017
	Collaborative learning environment that is based on exchanging comments among students.	Improvements in students’ performance.	Echeverría et al., 2017

#### Summary

Learning approaches offered different parameters that can be used to get insight into the behaviors of learners. Assessment and programming data were mainly used to identify learning behaviors. Programming specific behaviors can be examined through students’ programming activities. The parameters to analyze the programming behaviors can dynamically be acquired while solving the programming problems or can be obtained statically through the submitted code. The programming analysis was mainly performed for identifying the parameters that could more accurately be used to predict students’ course performance. In addition, the parameters to analyze the learning behaviors can be acquired by scrutinizing different learning styles and processes. The collaborative learning approaches demonstrated positive impact on students’ learning. Such approaches can be useful for managing large groups of students.

#### Tool

Tool-centric studies were further classified into IDE and support categories. The classification of IDE includes studies, which analyzed the impacts of using one or more features of IDE on students’ learning. Support tools were further categorized on the basis of the purposes these tools mainly serve or the types of support features that were examined. The sub-categories of support tools include: visualization, which visually demonstrates different aspects of IPC; prediction, which predicts students’ performance; personalized learning, which supports the learning processes on the basis of individual’s learning needs; and feedback, which assists in providing feedback to learners. Table 9 presents brief descriptions of the selected studies that examined different tools and the respective findings of these studies.

**Table 9:** Tool focused studies.

Leaf node categories	Brief description of major area of focus	Brief description of major findings	Articles
**IDE**			
Web-based	Identification of anti-patterns in students’ programs.	Identification of patterns showing better outcomes.	Ureel & Wallace, 2019; Ureel & Wallace, 2015
	Detection of changes in programming behavior to find students who need special assistance in programming.	Identification of students who need additional support to learn programming.	Estey, Keuning & Coady, 2017
	Effectiveness of web-based IDE.	Significant relationship between web-based programming tool and students' performance.	España-Boquera et al., 2017
	Integration of students’ programming activities.	Helped in reducing students’ problems.	Edwards, Tilden & Allevato, 2014
	Presence of non-terminating code through infinite loops.	Supported programming activities.	Edwards, Shams & Estep, 2014
**Support**			
Visualization	Code analysis to visualize working progress.	The tool provided visual analysis of differences between the codes.	Heinonen et al., 2014
Prediction	Peer programming feedback and adaptive learning to predict students’ performance.	The system was effective to support learning.	Azcona, Hsiao & Smeaton, 2018
	A Java grader system for performance prediction using machine learning algorithms.	The tool predicted performances by forecasting the final grades.	Koong et al., 2018
Feedback	Feedback by scrutinizing the students’ programs.	Auto- feedback on student codes to support learning.	Berges et al., 2016; Ureel & Wallace, 2019; Ureel & Wallace, 2015
	Feedback delivery of paper-based evaluation.	The system found effective in transmitting the feedback to students.	Hsiao, Huang & Murphy, 2017
	Feedback through graphs by examining the code.	No major difference in students’ performances without interactions.	Seanosky et al., 2017
Personalized learning	Scrutinizing the programming and learning behaviors to identify individual learning needs.	Supported students by recommending personalized learning material.	Fu et al., 2017
	Platform for self-paced learning.	Enhanced motivation for learning.	Su et al., 2015
	A system to support, motivate, and guide students by online reviewing their work.	The tool supported the process of learning by optimizing the learning efforts.	Hijon-Neira et al., 2014
	Analyzing the programming behaviors of students through tool interactions.	Identification of programming behaviors to design the personalized course activities.	Pereira et al., 2020

#### Summary

IDE focused studies investigated the enhanced features of different IDEs that could help in solving programming problems. These studies mainly examined the tools for the features, which were more sophisticated than the typical IDEs offer. The support tools were examined to assist one or more components of IPC. The tools were designed to support teaching through performance predictions. Instructors can optimize their teaching efforts through the support features of tools by auto-evaluation of the performance predictions and learning states of the learners. The support tools for learners were mostly used to deliver feedback and assist individual learning processes by evaluating the personalized learning needs of students. Auto-identification of parameters to precisely examine learning can help instructors improve the learning support. It can also be useful to design interventions as per the specific requirements of the learners.

#### Assessment

The assessment related aspects were further explored by analyzing assessment techniques and assessment material. The sub-categories of assessment techniques include grading and examination. The sub-category of grading includes the studies which examined the techniques of evaluating and ranking students according to their demonstrated understanding levels. The sub-category of examination covers studies in which the effectiveness of some assessment process is investigated. The assessment material is further classified into assessment instruments and assessment items. Table 10 illustrates brief descriptions of the selected studies that were focused on the aspects related to assessments and the associated findings of these studies.

**Table 10:** Assessment focused studies.

Leaf node categories	Brief description of major area of focus	Brief description of major findings	Articles
**Technique**			
Grading	Rule-based assessment to examine cognitive competency of students by applying Bloom’s taxonomy.	Identification of understanding levels of programming concepts.	Ullah et al., 2019
	Investigating the inconsistencies of grading.	Differences were found while grading same solutions from different graders.	Albluwi, 2018
Examination	Self-assessment technique for investigating the persistency in learning.	Consistent patterns of learning reflect better outcomes.	Chung & Hsiao, 2020
	Peer assessment technique to assess the students’ performance.	Peer assessment found to be a useful technique for assessing the large cohort of students.	King, 2018
	Peer review of students’ concepts maps to examine students’ understanding and higher level of thinking.	No improvements was identified in higher level thinking.	Turner, Pérez-Quiñones & Edwards, 2018
**Material**			
Assessment items	Assessment items ranking according to Bloom’s taxonomy and the application of the developed rubrics to rank the assessment items.	Identification of difficulties in learning that directed towards designing appropriate class activities.	Dorodchi, Dehbozorgi & Frevert, 2017
	Evaluation of assessment material to design future assessments.	Guide to develop effective assessment items.	Zur & Vilner, 2014
	Investigation of additive factors model to map knowledge into assessment items.	The model did not satisfactorily fit into IPC context.	Effenberger, Pelánek & Čechák, 2020
	Assessment items to examine students on different levels of thinking.	Higher precision attained in results.	Omer, Farooq & Abid, 2020
	Cue-based practical assessments.	Clarity of expected solution at students’ end.	Doshi, Christian & Trivedi, 2014
Assessment instruments	Fill in the blanks of programs, as an instrument to find students’ performance in programming.	Identified high correlation in students’ performance and the final outcomes.	Funabiki, Ishihara & Kao, 2016
	Relationship between hands-on exercises and final exams at various levels of cognition as per Bloom’s taxonomy.	Identification of dependencies among the written assessment and final scores.	Ahmad et al., 2017
	Examining the most suitable test for assessing students’ knowledge.	Students' performances were assessed though different types of assessment instruments.	Gomes, Correia & Abreu, 2016
	Max-min technique to design effective assessment.	Identification of parameters to design effective assessment.	Allinjawi, Al-Nuaim & Krause, 2014

#### Summary

Self and peer assessment techniques were identified as useful approaches of assessments. The appropriateness of assessment instruments to assess learners is a significant dimension to emphasize the quality of assessment. Some work has been performed to rank and grade learners at various cognitive levels; however, no specific guideline is identified to devise the rubrics for cognitive assessments. Generally, the studies assessed learning without providing information about the specific cognitive levels of learners on which the learners were assessed. The conventional assessment systems can be enhanced through the real-time analysis of learning. For this purpose, a process could be in place to identify the optimal set of parameters that can be used for dynamic analysis of students’ performance.

#### Content

The content focused studies were categorized into programming language and concepts. Programming language as a sub-category of content includes studies, which either presented comparison of programming languages or analyzed the learning difficulties related to specific programming languages. The sub-category of concepts presents studies in which different choices of constructs were compared to solve a programming problem. It also includes the studies in which specific IPC concepts were analyzed at various cognitive levels of learners. Table 11 enlists brief descriptions of the selected studies that were focused on IPC contents along with the respective findings.

**Table 11:** Content focused work.

Leaf node categories	Brief description of major area of focus	Brief description of major findings	Articles
**Concepts**			
Comparison	Difficulties students face in understanding various programming concepts.	Identification of threshold programming concepts.	Yeomans, Zschaler & Coate, 2019
	Choice of concepts to solve programming problem (recursion or iteration).	Identification of concept that was appropriately used to solved programming problem (iteration) .	Esteero et al., 2018
Cognition	Cognitive learning in programming loops.	Identification of students who face difficulties in understanding loops.	Gomes & Correia, 2018
	Students’ understanding of objects and classes.	Deep analysis of misconceptions in objects and classes.	Xinogalos, 2015
**Programming language**			
Comparison	Comparison of Python and C to check the impact of programming language on students’ performance.	Python presented better learning outcomes than C.	Wainer & Xavier, 2018
Difficulty	Addressing the difficulties of programming through mixed languages.	Motivation to learn programming was increased.	Santana, Figueredo & Bittencourt, 2018

#### Summary

The cognitive analysis of programming concepts was performed by applying the Bloom’s taxonomy. Analysis of specific concepts was performed for various IPC concepts, such as loops, recursion, iteration, objects, and classes. It appears that the choice of programming languages could impact the learning processes. Hence, a comprehensive study to analyze the effect of teaching different programming languages on students’ performance could be useful in order to identify the most appropriate programming language to teach IPC. In this context, a study has been conducted to evaluate the first programming languages, which presented Java as the most appropriate programming language to teach IPC (Farooq et al., 2014). In addition, utilizing some systematic approach to analyze first programming languages could help in choosing an appropriate programming language out of the available choices. One of the efforts in this direction has been made in which the authors proposed a framework for evaluating the first programming languages (Farooq, Khan & Abid, 2012).

### RQ3: What contributions can be perceived on the basis of potential benefits or impacts of IPC research in the field?

Table 12 presents the classification with respect to the perceived contributions of the selected studies in the field. About 30% of the selected studies contributed towards approach examination by evaluating different teaching, learning, and analysis approaches. Another major contribution has been made through features inspection, which was reflected in about 29% of the selected studies. The aspects automation was posed in about 17% of these studies, and the intricacies identification was shown in about 12%, while the group classification was implied in about 12 % of the selected studies.

**Table 12:** Perceived contributions of IPC research.

Categories	Studies		
Features inspection	McCall & Kölling, 2019; Azcona, Hsiao & Smeaton, 2018; Pereira et al., 2020; Chaweewan et al., 2018; Omer, Farooq & Abid, 2020; Ninrutsirikun et al., 2020; Premchaiswadi, Porouhan & Premchaiswadi, 2018; Turner, Pérez-Quiñones & Edwards, 2018; Ahadi, Hellas & Lister, 2017; Ahmad et al., 2017; al-Rifaie, Yee-King & d’Inverno, 2017; Carter, Hundhausen & Adesope, 2017; Castro-Wunsch, Ahadi & Petersen, 2017; Chung & Hsiao, 2020; Funabiki, Ishihara & Kao, 2016; Watson, Li & Godwin, 2014; Ahadi et al., 2015; Ashenafi, Riccardi & Ronchetti, 2015; Carter, Hundhausen & Adesope, 2015		
Approach examination	Liao et al., 2019; King, 2018; Lagus et al., 2018; Lin et al., 2018; Santana, Figueredo & Bittencourt, 2018; Effenberger, Pelánek & Čechák, 2020; Echeverría et al., 2017; España-Boquera et al., 2017; Iqbal Malik & Coldwell-Neilson, 2017; Seeling & Eickholt, 2017; Malliarakis, Satratzemi & Xinogalos, 2016; Seeling, 2016; Hilton & Rague, 2015; Janke, Brune & Wagner, 2015; Rubio et al., 2014; Rosiene & Rosiene, 2015; Scott et al., 2015; Wood & Keen, 2015; Doshi, Christian & Trivedi, 2014; Edwards, Tilden & Allevato, 2014		
Intricacies identification	Yeomans, Zschaler & Coate, 2019; Albluwi, 2018; Gomes & Correia, 2018; Delev & Gjorgjevikj, 2017; Dorodchi, Dehbozorgi & Frevert, 2017; Berges et al., 2016; Simkins & Decker, 2016; Allinjawi, Al-Nuaim & Krause, 2014		
Group classification	Ullah et al., 2019; Esteero et al., 2018; Wainer & Xavier, 2018; Estey, Keuning & Coady, 2017; Kumar, 2017; Gomes, Correia & Abreu, 2016; Zur & Vilner, 2014; Xinogalos, 2015		
Aspects automation	Ureel & Wallace, 2019; Bhatia, Kohli & Singh, 2018; Koong et al., 2018; Fu et al., 2017; Hsiao, Huang & Murphy, 2017; Seanosky et al., 2017; Su et al., 2015; Ureel & Wallace, 2015; Edwards, Shams & Estep, 2014; Heinonen et al., 2014; Hijon-Neira et al., 2014		

**Table 13:** Quality scoring of the shortlisted studies.

Ref. No	(a)	(b)	(c)	(d)	Total
Pereira et al., 2020	1	1	1	2	5
Omer, Farooq & Abid, 2020	1	1	1	1.5	4.5
Chung & Hsiao, 2020	0.5	1	1	0	2.5
Effenberger, Pelánek & Čechák, 2020	1	0.5	1	0	2.5
Ninrutsirikun et al., 2020	1	1	1	0	3
Liao et al., 2019	1	1	1	2	5
McCall & Kölling, 2019	0.5	1	1	2	4.5
Ullah et al., 2019	0.5	1	1	2	4.5
Ureel & Wallace, 2019	1	1	1	1	4
Yeomans, Zschaler & Coate, 2019	1	1	1	2	5
Albluwi, 2018	0.5	1	1	2	4.5
Azcona, Hsiao & Smeaton, 2018	1	1	1	0.75	3.75
Bhatia, Kohli & Singh, 2018	1	1	1	1.5	4.5
Chaweewan et al., 2018	1	1	0.5	0	2.5
Esteero et al., 2018	0.5	1	1	1	3.5
Gomes & Correia, 2018	0.5	1	1	0.75	3.25
King, 2018	0.5	1	1	0.75	3.25
Koong et al., 2018;	1	1	1	0.75	3.75
Lagus et al., 2018	1	0.5	1	2	4.5
Lin et al., 2018	0.5	1	1	2	4.5
Premchaiswadi, Porouhan & Premchaiswadi, 2018	1	1	1	0.75	3.75
Santana, Figueredo & Bittencourt, 2018	0	1	1	0.75	2.75
Turner, Pérez-Quiñones & Edwards, 2018	0.5	1	1	2	4.5
Wainer & Xavier, 2018	0.5	1	1	2	4.5
Ahadi, Hellas & Lister, 2017	0.5	1	1	2	4.5
Ahmad et al., 2017	0.5	1	1	0	2.5
al-Rifaie, Yee-King & d’Inverno, 2017	0.5	1	1	0	2.5
Carter, Hundhausen & Adesope, 2017	1	1	1	2	5
Castro-Wunsch, Ahadi & Petersen, 2017	1	1	1	1	4
Delev & Gjorgjevikj, 2017	0.5	1	1	0	2.5
Dorodchi, Dehbozorgi & Frevert, 2017	0.5	1	1	0.75	3.25
Echeverría et al., 2017	0.5	1	1	2	4.5
España-Boquera et al., 2017	0.5	1	1	0.5	3
Estey, Keuning & Coady, 2017	0.5	1	1	1	3.5
Fu et al., 2017	1	1	1	0	3
Hsiao, Huang & Murphy, 2017	0.5	1	1	2	4.5
Iqbal Malik & Coldwell-Neilson, 2017	1	1	1	2	5
Kumar, 2017	0.5	1	1	0.75	3.25
Seanosky et al., 2017	0.5	1	1	0.75	3.25
Seeling & Eickholt, 2017	0.5	1	1	0.75	3.25
Berges et al., 2016	0.5	1	1	0	2.5
Funabiki, Ishihara & Kao, 2016	0.5	1	1	0	2.5
Gomes, Correia & Abreu, 2016	0.5	1	1	0.75	3.25
Malliarakis, Satratzemi & Xinogalos, 2016	0.5	1	1	2	4.5
Seeling, 2016	0.5	1	1	0.75	3.25
Simkins & Decker, 2016	0	1	1	0.75	3
Ahadi et al., 2015	0.5	1	1	0.75	3.25
Ashenafi, Riccardi & Ronchetti, 2015	1	1	1	0.75	3.75
Carter, Hundhausen & Adesope, 2015	1	1	1	0.75	3.75
Hilton & Rague, 2015	0.5	1	1	0.75	3.25
Janke, Brune & Wagner, 2015	0	1	1	1.5	3.5
Rosiene & Rosiene, 2015	0.5	1	1	0.75	3.25
Scott et al., 2015	0.5	1	0.5	2	4
Su et al., 2015	1	1	1	0.75	3.75
Ureel & Wallace, 2015	1	1	1	0.75	3.75
Wood & Keen, 2015	0.5	1	1	1	3.5
Xinogalos, 2015	0.5	1	1	2	4.5
Allinjawi, Al-Nuaim & Krause, 2014	0.5	1	1	2	4.5
Doshi, Christian & Trivedi, 2014	0.5	1	1	0	2.5
Edwards, Shams & Estep, 2014	0.5	1	1	1	3.5
Edwards, Tilden & Allevato, 2014	0.5	1	1	1	3.5
Heinonen et al., 2014	0.5	1	1	1	3.5
Hijon-Neira et al., 2014	1	1	1	0.75	3.75
Rubio et al., 2014	0.5	1	1	0.75	3.25
Watson, Li & Godwin, 2014	0.5	1	1	1	3.5
Zur & Vilner, 2014	0.5	1	1	0.75	3.25

The approaches were examined to evaluate the usefulness of different techniques, such as collaborative learning and peer instructional techniques (Liao et al., 2019; King, 2018). Moreover, the effect of changing the sequence of teaching different programing concepts was investigated (Janke, Brune & Wagner, 2015). In addition, a transfer learning approach was evaluated for examining the improvements in prediction accuracies (Lagus et al., 2018). This approach was proposed to manage the heterogeneity of data by assigning weights to the instances that were involved in predictions. The learning approaches were examined, e.g., through different learning patterns (Echeverría et al., 2017), and by applying the genetic algorithms for identification of personalized learning needs (Lin et al., 2018). Gamification was explored as a technique to enhance students’ programming abilities (Malliarakis, Satratzemi & Xinogalos, 2016).

The features were inspected to evaluate the parameters that can potentially be used to examine students’ performance. Studies, in this category, examined the relationship between students’ interaction with tools and their performance in exams. In this context, the features that were evaluated include the number of actions performed, access time, and the frequency of using one or more sections of tools (Premchaiswadi, Porouhan & Premchaiswadi, 2018; Ahadi, Hellas & Lister, 2017). Moreover, the changes in the compilation states were also considered to determine the students’ performance (Carter, Hundhausen & Adesope, 2015; Carter, Hundhausen & Adesope, 2017). Furthermore, the static or gradually changing traits of students, such as gender, past programming experience, aptitude, skills, learning styles, and academic records, were evaluated (Chaweewan et al., 2018). Additionally, the dynamically changing factors that can be identified from programming behaviors, were also scrutinized (Castro-Wunsch, Ahadi & Petersen, 2017). Similarly, the cognitive and non-cognitive factors were analyzed for identifying the students’ performance (Ninrutsirikun et al., 2020).

The automation of aspects was performed to support the course related processes. This includes studies, which supported the learning and teaching processes through automated feedback deliverance (Ureel & Wallace, 2015; Seanosky et al., 2017). Moreover, the automation of coding evaluation was performed to provide detailed insight into the quality of code by identifying the parameters like non-terminating code (Edwards, Shams & Estep, 2014) and anti-patterns (Ureel & Wallace, 2019) in students’ programs.

The intricacies were identified to find areas that need specific attention for potential improvements, e.g., by evaluating the difficulties in learning specific programming language or concept (Yeomans, Zschaler & Coate, 2019; Gomes & Correia, 2018). Similarly, the difficulties in grading were observed when same solutions were evaluated from different graders (Albluwi, 2018).

The group classification was performed, e.g., to categorize the assessment items according to different levels of cognition (Ullah et al., 2019), and identify the appropriate assessment types to evaluate learning (Gomes, Correia & Abreu, 2016). It further covers the research, which segregated the groups of learners according to different performance levels (Estey, Keuning & Coady, 2017).

## Discussion and analysis

### Principal findings

The existing work of IPC review mostly examined teaching and learning, which have been identified as significant dimensions of IPC research. However, our findings reveal that a wide range of work is centralized to tools, which are developed for supporting the related course processes. In this context, the feedback and personalized learning tools presented the major share of the tool-centric research in IPC. Moreover, our analysis indicated that less work is performed to present the frameworks and models as solutions of the specified research problems. In addition, the IPC research has mainly contributed by evaluating different factors that could determine the performance of learners. This leads to the exploration of diverse ways of assessing learners in addition to following the conventional assessment approaches. Furthermore, this review emphasizes the analysis of programming behavior through more methodical techniques in order to gain a better insight into the learning needs of IPC students.

### Future implications

The future implications are presented by listing some advices for IPC instructors and open research issues for IPC researchers.

#### Advice for instructors

As a result of synthesizing the selected studies, some effective practices of conducting IPC have been identified. These practices are listed as advices for instructors. The instructors of IPC can consider these advices to improve the quality of the related aspects of the course.

- *Prediction-based intervention design:* The difficulty levels of programming concepts tend to rise in the later stages of conducting IPC. Therefore, making use of some performance prediction measures can be beneficial to design and deliver appropriate interventions before teaching the complex concepts of IPC (Omer, Farooq & Abid, 2020).
- *Cognitive assessment:* Assessing learners on different cognitive levels can be useful for accurate evaluations of learning (Dorodchi, Dehbozorgi & Frevert, 2017). Moreover, assessing the cognitive processes through the exercises enforcing computational thinking can also serve to identify the specific cognitive gaps of leaners (Rojas-López & García-Peñalvo, 2018).
- *Gamification as learning approach:* Use of games has emerged as an essential technique to support learning in IPC (Malliarakis, Satratzemi & Xinogalos, 2016). This can be an effective approach to develop students’ interests in programming.
- *Collaborative learning platforms:* Facilitating collaborative environments by establishing the peer learning platforms turned out to be a useful practice in IPC (Ashenafi, Riccardi & Ronchetti, 2015; Echeverría et al., 2017). Tool-supported collaborations could be effective to aid the learning process of novice programmers.
- *Use of web-based IDE*: Web-based IDE can be useful for programming as it could help students to work remotely on programming problems (España-Boquera et al., 2017; Seeling & Eickholt, 2017). In addition, students’ learning progress can also be conveniently examined by instructors through the submitted programs.
- *Tool-assisted feedback:* Feedback deliverance can be improved by using tools. The tool-assisted feedback could support the process of identifying the learning gaps of students and help them to understand their personalized learning needs (Fu et al., 2017).
- *Grading consistencies and precisions:* A study identified huge differences in grading when the same solutions of programming problems were evaluated by different graders (Albluwi, 2018). Applying uniform marking schemes could help to achieve consistencies in grading specifically in case of having more than one graders within or across the terms. Moreover, as a programming problem needs to be solved by applying different concepts, the distribution of marks among various concepts involved in solving a given problem could improve precisions in grading.
- *Transparency in assessment:* Assessment would be less effective if the students remain unclear about the aspects to be emphasized while solving a given programming problem. Revealing some aspects of assessment criteria to students can help them to differentiate the major and minor aspects that are inquired in assessment items. Another way to address this concern is to provide cues with the problem statements (Doshi, Christian & Trivedi, 2014). However, care must be taken about providing the level of information in the cues, which should not question the assessment process.
- *Utilization of tool-interaction data:* The tool-interaction data is identified as one of the prominent parameters to gage students’ involvements in the course (Chaweewan et al., 2018). Analysis of tool-interaction data could be useful to understand students’ learning behaviors. Similarly, the analysis of programming data related to the use of tools can also help to get insight into the individual progression of learning.

#### Open research issues

The analysis of the findings revealed some significant dimensions that can be considered for future research of IPC. These are listed as open research issues of IPC.

- **Robustness of IPC research:** The findings of most of the selected studies were based on particular cohorts of students. Rare efforts are observed to examine the robustness of existing work by replicating the same scenarios on different groups of learners. A study performed replication analysis of the research conducted in programming courses and found differences in the results (Ihantola et al., 2015). This gap questions the applicability of studies on similar scenarios and indicates the need of investigating the robustness of IPC research.
- **Code testing:** Generally, the IPC contents do not include the topic of testing. Consequently, students would not be able to formally verify or validate their programs. The findings of the studies, which analyzed learning approaches through programming related parameters like errors (McCall & Kölling, 2019) and test cases passed (Ureel & Wallace, 2015), could have been influenced due to this potential gap in the IPC contents. A recent research explored different ways of teaching software testing in IPC (Scatalon, Garcia & Barbosa, 2020); however, the identification of appropriate level of cognition to teach the concept of code testing in IPC is an area that still needs to be examined.
- **Course structuring:** Assessment of learning at initial stages of conducting IPC, and analyzing the impacts of the outcomes of assessment on forthcoming stages can help in early identification of learning gaps. To follow such practice, IPC needs to be structured by linking various stages of course covering different concepts. This can be done by using the technique of concept mapping. A study analyzed the use of concept mapping in computer science courses and identified the increase in diversity of its use in programming courses (Santos et al., 2017). A recent work proposed the use of concept mapping technique to examine cognitive performance on higher level programming concepts (Omer, Farooq & Abid, 2020). However, the use of concept mapping can further be evaluated from various dimensions like investigating the cognitive learning patterns, differentiating the cognitive patterns of specific groups of learners, and examining the threshold concepts.
- **Behavioral assessment:** The behavioral assessment supports precision teaching, which is a constructional approach to solve behavioral and learning problems (Evans, Bulla & Kieta, 2021). IPC generates huge volumes of data in the form of submitted programs, and tool interactions of students, which can be utilized for assessing the learning behaviors of IPC students. The behavioral assessment can also be used for developing and maintaining the programming profiles in order to track the behavioral progressions of novice programmers.
- **Gamification:** An increasing trend of using gamification, in computer science education, has been observed in recent years (Ahmad et al., 2020). Gamification with mobile-assisted learning can enhance the intrinsic motivation of learners (Ishaq et al., 2021). A study suggests that the use of games can improve students’ engagements in programming (Rojas-López et al., 2019). However, rare work is observed to evaluate how gamification can precisely be used to support teaching, learning, and assessment of specific IPC concepts. Moreover, designing and implementing games as learning objects with respect to certain cognitive levels of learners is an area that still needs attention of the community.

## Limitations

Main limitations related to this review are listed below:

- A possibility of bias exists in the selection of studies due to the subscription limitations of our university library, which was the main source of extracting the papers from the digital repositories. However, it was managed by acquiring the relevant papers through other institutes having different subscription packages.
- The classifications on the basis of areas of focus and perceived contributions were carried out by considering the aspects that were most related to the investigating areas of this work. The overlapping in the classification can exist, which has been discussed while presenting the results. It was further managed by reviewing the classification of the selected studies three times.
- The selection and classification of studies were performed by the authors and reviewed by two independent reviewers to minimize the risk of any bias. The results of these tasks were compared and the discrepancies were discussed until the consensus was reached. The Kappa coefficient (McHugh, 2012) was measured to evaluate the interrater reliability. The value of the Kappa coefficient (McHugh, 2012) was 0.86, which depicts strong agreement (Landis & Koch, 1977) among the authors.
- A number of different keywords were used to find the most relevant papers. However, there exists a possibility that some studies used additional or alternate keywords due to which some papers may have overlooked.
- The quality appraisal criteria of the selected studies were based on a research conducted in the relevant field (i.e., computer science education). It was opted to mitigate the risk of any bias in the quality assessment of the included studies.

## Conclusions

This study has been conducted to examine the recent advancements in IPC. After carefully evaluating the papers searched from prominent research portals, 66 articles were shortlisted for conducting this review. The shortlisted articles were synthesized on the basis of bibliometric facts, types of research, areas of focus, and perceived contributions. Based on in-depth analysis of the selected studies, this review proposed a taxonomy of IPC that presents 23 different dimensions of IPC research. This work contributes in the field by classifying and examining the state-of-the-art research in IPC, and highlighting the principal findings identified by reviewing the IPC research. Furthermore, as the main objective of this work was to assist IPC instructors and researchers in their respective domains, we concluded this work by presenting the future implications, which highlighted the advices for instructors and the open issues for IPC researchers. The identified gaps indicated that very few methodical approaches have been proposed to examine the different components of IPC. Moreover, the cognitive assessments could improve the precision in assessments by ranking learners at various cognitive levels. Therefore, it can be concluded that the structuring of course contents could help in identifying the aspects that effect the students’ performance. Another interesting future research direction suggests to utilize the huge volumes of data that are mainly generated as a result of the students’ interactions with tools. This can help to get insight into students’ learning states and design optimized interventions for novice programmers.

## Additional Information and Declarations

### Data Availability

The following information was supplied regarding data availability: This work is a literature review.
